# Scientific slop: generative AI, research incentives, and the integrity of the genomic knowledge base

**DOI:** 10.3389/fgene.2026.1894137

**Published:** 2026-10-28

**Authors:** Dov Greenbaum

**Affiliations:** 1 Department of Molecular Biophysics and Biochemistry, Yale University, New Haven, CT, United States; 2 Zvi Meitar Institute for Legal Implications of Emerging Technologies, Reichman University, Herzliya, Israel; 3 Harry Radzyner Law School, Reichman University, Herzliya, Israel; 4 Berkeley Law, University of California, Berkeley, Berkeley, CA, United States

**Keywords:** AI governance, AI slop, data provenance, future of scientific research, peer review, research integrity

## Abstract

Artificial intelligence is being integrated into genetics and genomics at a pace that outstrips existing governance mechanisms. This article identifies a structural threat: AI systems operating within the incentive architecture of scientific publishing face systematic pressure to optimize for publishability rather than truth, producing outputs that may be internally coherent yet empirically untethered. It traces three layers of integrity failure—inadvertent contamination through hallucination, deliberate fraud made cheaper and harder to detect, and epistemic blurring, the progressive loss of provenance that erodes transparency. It examines how AI enables forms of virtual experimentation that existing ethics frameworks do not clearly govern. It assesses science’s principal self-governance institutions and argues that each is structurally mismatched to AI-generated epistemic risk. The article proposes infrastructure-embedded governance: accountability mechanisms built into databases, repositories, journals, indexing platforms, and funding pipelines, including system-level retractability, credibility ledgers, auditable computational notebooks, training-data conflict-of-interest disclosure, sanctionability, trust-weighted indexing, reproducibility metrics, replication incentives, governance-weighted journal assessment, and controlled epistemic stress-testing. The central argument is that governance must target the infrastructure layer and realign scientific incentives so that trustworthiness is competitively rewarded before recursive contamination becomes self-reinforcing.

## 1 Introduction: AI, genomic science, and the integrity of the knowledge base

This article argues that AI’s integration into genetics poses a structural threat to the field’s epistemic infrastructure. Not because the technology is defective, but because it is absorbed into an environment that already privileges publishability over validity. The consequences manifest across three layers: inadvertent contamination, deliberate fraud made cheaper and harder to detect, and epistemic blurring, the progressive loss of provenance that renders the origin and epistemic status of a claim untraceable across the knowledge base. AI also enables virtual experimentation that, as described herein, is not clearly governed by existing ethics frameworks. The article assesses science’s self-governance institutions, identifies structural mismatches between their existing oversight mechanisms and AI-mediated epistemic risks, and proposes infrastructure-embedded governance targeting the knowledge flows through which contamination propagates.

**FIGURE 1:**
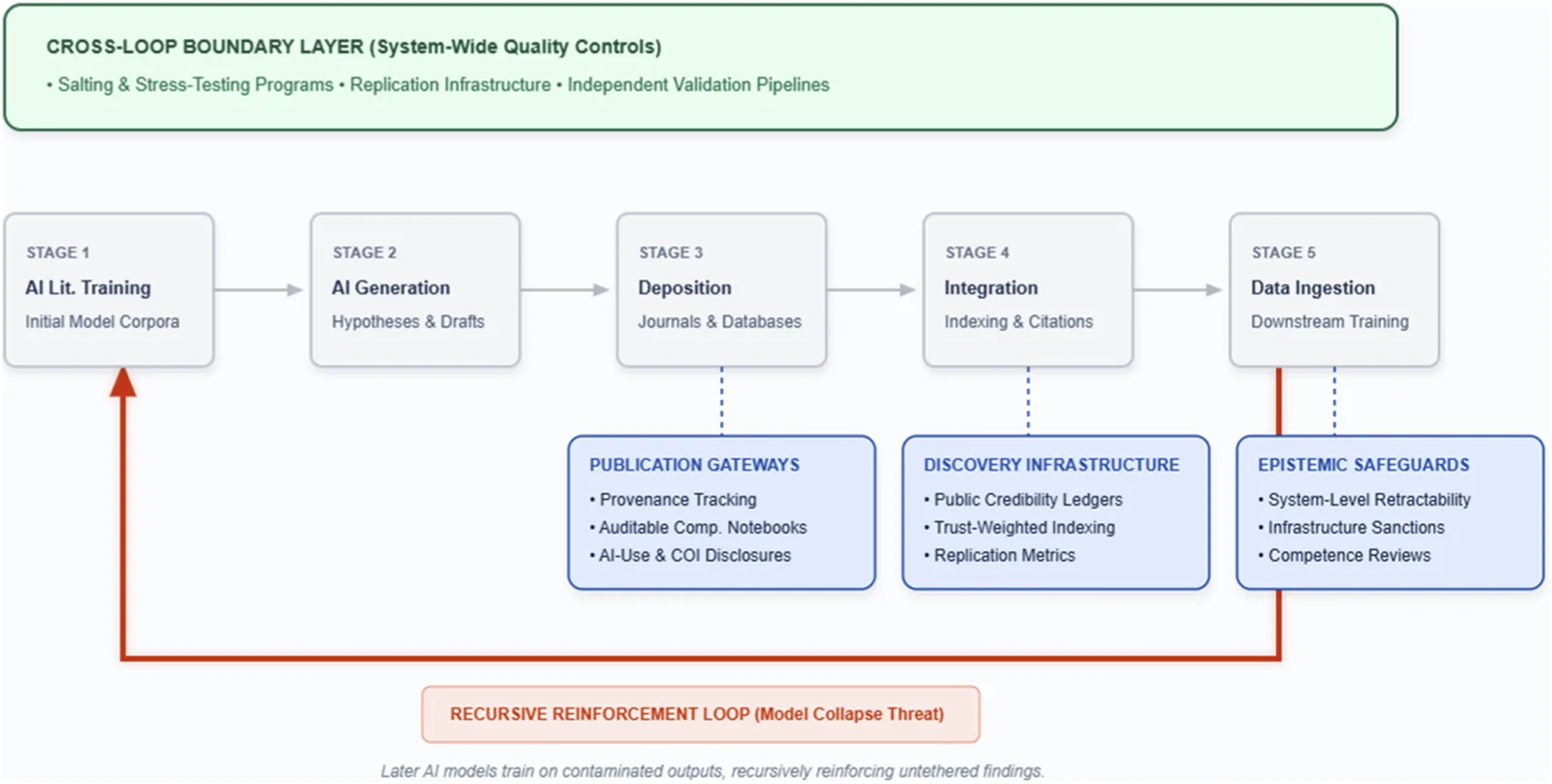
Recursive contamination and governance intervention points in AI-mediated genomic science.

This article contributes to the 15th anniversary research Topic of *Frontiers in Genetics* by addressing what may become the defining ELSI challenge for the field’s next decade. AI-generated knowledge is entering the databases, annotation pipelines, and publication systems on which every genetics specialty depends. If that shared infrastructure is compromised, the downstream consequences for variant classification, drug-target identification, population genomics, and clinical translation will extend across every section represented in this volume. The ELSI challenge is not adjacent to the scientific agenda. It is a precondition for it.

To be clear at the outset: AI is among the most powerful tools ever introduced into the life sciences, and its continued integration is both inevitable and, on balance, enormously beneficial. AI-driven variant classification and prioritization are increasingly being used to accelerate rare-disease diagnosis, offering a path to earlier answers for patients who might otherwise need to wait for years. Computational methods are increasingly being used to identify drug-repurposing candidates for rare diseases with limited commercial investment; detect patterns across large genomic, clinical, and imaging datasets; and accelerate parts of the discovery and diagnostic workflow (Abdallah et al., 2023; Nishat et al., 2025; Kara et al., 2026). The field cannot do without these capabilities, nor should it try.

The argument of this article is not that AI in genetics is dangerous and should be constrained. It is that AI in genetics is so valuable and will become so deeply embedded in the field’s epistemic infrastructure that the consequences of getting governance wrong are correspondingly severe. A tool this powerful, operating at this scale, within an incentive structure already known to reward publishability over validity, requires governance proportional to both its promise and its risk. The proposals advanced here are designed to protect AI’s scientific value, not to limit it: infrastructure that ensures AI-generated knowledge is trustworthy is infrastructure that keeps AI usable.

### 1.1 From instrument to agent

Science has always delegated to instruments: telescopes extended perception, statistics extended inference, and computation extended analysis. Each step moved researchers further from the raw phenomenon and closer to supervision. A modern geneticist no more reads individual base pairs than a modern astronomer grinds lenses. The trajectory has been largely unidirectional, and efforts to reverse it have rarely succeeded.

The 1975 Asilomar Conference (Berg et al., 1975) remains the canonical example of prospective scientific self-governance, but its containment framework was relaxed within a decade as competitive pressures mounted (Krimsky, 2005). The broader record confirms that technological restraint succeeds only where substitutes exist, competitive incentives to defect are weak, or regulation can target a discrete chokepoint. Where none holds, such as in nuclear proliferation, gain-of-function research (Giordano and DiEuliis, 2025), or stem-cell tourism (Chodosh and Lisher, 2003; Brownsword, 2004; Xiao et al., 2026), restrictions prove partial, temporary, or jurisdictionally bounded. AI satisfies none of these conditions: no substitute exists, adoption incentives are immediate, and no chokepoint is available because the technology is general-purpose and its outputs enter distributed infrastructure upon publication.

Artificial intelligence also differs from its predecessors in a critical respect: it extends/replaces cognition itself. Rather than amplifying perception or calculation, AI generates hypotheses, interprets results, identifies patterns across datasets too large for human comprehension, and increasingly designs the experiments to test its own predictions (Gottweis et al., 2026; Xie et al., 2025; Xiong et al., 2026). Arguably, the scientific community has embraced this shift not reluctantly but enthusiastically (Resnik et al., 2026). This embrace is now visible at scale: AI-related natural-science publications increased almost 30-fold between 2010 and 2025, and more than 80,000 natural-science publications in 2025 mentioned AI (Jones, 2026).

### 1.2 The AlphaFold precedent

No development illustrates this embrace more clearly than AlphaFold. DeepMind’s protein-structure prediction system was adopted by researchers across the planet (Kovalevskiy et al., 2024), cited nearly 50,000 times (Jumper et al., 2021), and rewarded with the 2024 Nobel Prize in Chemistry. No ethics panel asked whether protein-structure prediction should remain a human task. The community’s value structure was revealed with unusual clarity: scientists valued the result, not the process. If AI can deliver an answer faster, cheaper, and with comparable accuracy, adoption follows. Governance, if it arrives at all, arrives after the fact.

AlphaFold established the template for later scientific AI. Systems for variant classification, drug-target identification, literature synthesis, experimental design, and autonomous hypothesis generation will be judged by the same calculus: not whether the task should remain human, but whether the system produces useful outputs quickly and efficiently enough to justify adoption.

By 2025, AlphaFold had become infrastructure: integrated into biochemical workflows, graduate training, disease research, and drug-discovery pipelines (Kahn, 2025). Successful scientific AI becomes infrastructure before governance catches up. AlphaFold 3 complicated this model by initially restricting access and then releasing code only after community pressure (Offord, 2024; Google DeepMind, 2024). What provoked pushback was not delegation of cognition but the threat to reproducibility when corporate ownership limited the availability, revealing which values the community will defend.

However, the deeper asymmetry is evidentiary. AlphaFold’s predictions can be checked against experimentally determined structures (Abramson et al., 2024). As AI moves toward hypothesis generation, experimental design, and independent publication, the verification capacity diminishes at each step (Zhu M., et al., 2025; Son et al., 2025; Xie et al., 2025). Verification cannot be displaced by plausibility. Plausibility is what remains when verification has been priced out (Greenbaum, 2009). The community’s adoption enthusiasm will not diminish as verifiability declines; the gap between the two will be among the decade’s most significant ELSI challenges. Zenil et al. (2026) reinforced the point: closed-loop AI systems risk epistemic collapse without graded autonomy anchored to verifiable mechanisms.

### 1.3 AI as co-scientist: the current state

The transition from AI-as-instrument to AI-as-agent accelerated in 2025 and 2026. The shift is not that AI assists with discrete tasks but that emerging systems now participate in the formation of scientific inquiry itself: generating hypotheses, proposing experimental designs, ranking explanations, synthesizing literature, and refining research directions.^1^

Google DeepMind’s Co-Scientist uses specialized agents to generate hypotheses, design experiments, and synthesize evidence while keeping scientists in the loop (Gottweis et al., 2026). Stanford researchers used it to identify drug-repurposing candidates for liver fibrosis (Guan et al., 2025); Imperial College reported that it reproduced in days a hypothesis that took years conventionally (Penadés et al., 2025). These examples remain preliminary, but the direction is clear: AI is moving from analysis of outputs to participation in reasoning.

Other systems push the same trajectory further. FutureHouse’s Robin combines agentic AI with an iterative lab-in-the-loop workflow, in which computational reasoning is linked to experimental testing and feedback (Ghareeb et al., 2026; Ledford, 2026). More broadly, agentic AI, laboratory robotics, and real-time data integration are beginning to converge into closed-loop discovery systems (Zhang et al., 2025; Xiong et al., 2026). In such systems, the human role may shift from direct investigator to supervisor, auditor, or approver of machine-generated research cycles.

Current AI co-scientists remain dependent on human framing, validation, and judgment. A Nature editorial cautioned that humans remained structurally necessary and that the relationship between computational efficiency and genuine insight remains unproven (Nature, 2026). Zenil et al. (2026) warned that validation, interpretability, and human–AI coordination may become the effective bottlenecks. The question is not whether AI can generate more hypotheses but whether the resulting claims can be validated and integrated without degrading the record’s reliability.

DeepScientist illustrates the pattern: framing discovery as goal-driven optimization, it generated more than 5,000 ideas, tested approximately 1,100, and produced 21 innovations (Weng et al., 2025). The bottleneck was not imagination but filtering, testing, and validation, precisely where governance pressure will concentrate.

Multi-agent ‘virtual lab’ systems reinforce the point: agent teams that search, critique, rank, and synthesize hypotheses, sometimes producing reports resembling lab-meeting deliberations (Jones, 2025), demonstrate how quickly scientific work is shifting from individual cognition to AI-mediated group reasoning.

The current state, then, is transitional. AI co-scientists are not yet independent scientists in any meaningful institutional or moral sense. However, they are no longer mere instruments either. They occupy an intermediate position: systems that can shape the direction, pace, and evidentiary structure of research while remaining dependent on human institutions for validation and legitimacy. That intermediate status is precisely what makes them difficult to govern.

### 1.4 The positive case: what AI does well and what we would lose without it

AI-driven variant classification can identify pathogenic variants in hours. For a child with an undiagnosed rare disease, such tools may affect not merely the speed of analysis but whether a diagnosis is reached at all. AI co-scientists can identify drug-repurposing candidates for neglected diseases lacking commercial incentive (Adam, 2026). The scale of modern genomic data has exceeded human analytical capacity: population-scale gene–environment interactions, polygenic contributions, and epistatic effects across thousands of loci depend on computational methods no human team could replicate (Agrawal et al., 2026; Xiong et al., 2026). The positive case disciplines the governance argument: the goal is not to slow adoption but to ensure AI-generated knowledge is trustworthy enough to act on. Evidence from drought science confirms the pattern: Ascenso et al. (2026) found rapid AI growth but persistent weaknesses in explainability and data sharing. Adoption alone does not produce maturity. The governance proposed in this article is designed to protect that value, not constrain it.

This democratization promise, however, creates a tension that governance must address rather than ignore. AI tools offer the greatest relative productivity gains in settings where human expertise is scarcest, meaning that researchers and clinicians in low- and middle-income countries have the strongest rational incentive to adopt them and the least institutional capacity to evaluate their outputs independently. The policing infrastructure proposed in this article, credibility ledgers, provenance tracking, and trust-weighted indexing, operates on outputs regardless of where they originate; a hallucinated variant classification deposited in ClinVar is subject to the same curation process whether generated in Boston or Nairobi.

However, consequences of governance failure are most severe where human backup is the thinnest. A clinician relying on an AI variant classification as the only interpretive resource cannot cross-check it against local expertise that does not exist. Governance must be implementable across institutional contexts, not only at well-resourced centers. If compliance requires computational infrastructure that resource-constrained institutions lack, governance becomes a barrier to participation, and the democratization case collapses into its opposite. The goal is standards that travel: machine-readable, lightweight, and useful where the need is the greatest.

Clinical trust presents a related challenge. Clinicians may continue to rely on erroneous AI recommendations even when contrary evidence emerges (Vinas et al., 2026), and outcomes depend not only on model performance but also on how human and AI judgments are coordinated (Papale et al., 2026). Accordingly, governance should promote calibrated reliance rather than trust alone. Benchmark accuracy is also insufficient: clinical utility depends on calibration, uncertainty, relevant decision thresholds, external validation, and performance in actual clinical settings (Cabitza et al., 2026).

### 1.5 The impossible made possible: digital twins, virtual biology, and the ethics of experiments we never could run

AI is creating new categories of inquiry. Digital twins integrating genome, transcriptome, metabolome, microbiome, and sensor data could enable genuinely individualized medicine (Xiong et al., 2026; Zhang et al., 2025; Yu, 2026). Regulatory frameworks are adapting: the FDA Modernization Act 2.0 authorizes *in silico* approaches as part of the evidentiary basis for drug safety (Ahmed et al., 2023; Karanasiou et al., 2025; Chen B. et al., 2026). If the platforms capable of running digital twins remain proprietary, the mechanisms of human health risk becoming black-boxed corporate assets.

The same capability enables experiments never conducted because they could not be ethically justified. Virtual populations could be subjected to *in silico* pathogen exposure, eugenic selection scenarios, or ancestry-differentiated agent testing, computational analogs of what Nuremberg, Helsinki, and the Common Rule were created to prevent. Four concerns arise: (1) such knowledge is identical in dual-use content regardless of the method (Amram et al., 2023; Greenbaum, 2025); (2) digital twins implicate the consent and privacy of data subjects; (3) ethical limits maintain normative boundaries that virtual experimentation erodes by making the previously unthinkable routine; (4) AI co-scientists may preferentially gravitate toward virtual experimentation because of its speed, scalability, and reduced dependence on subject recruitment, potentially allowing ethically consequential research questions to migrate outside conventional human-subject review structures (Khosrowi, 2026; Tong et al., 2026).

Yet, the absence of conventional human subjects should not automatically eliminate ethical oversight. Review may remain appropriate where virtual experiments involve identifiable or re-identifiable genomic data, ancestry-specific analyses, clinically consequential predictions, or material dual-use risks. Oversight should, therefore, turn on the risks created by the data, inference, and application rather than simply whether participants are physically enrolled.

A related governance gap concerns AI training data sourced from the genomic literature. AI systems trained on published articles ingest not only findings but patient-derived data summaries, case descriptions, and population-level datasets whose consent frameworks did not contemplate machine-learning use. Commercial AI developers already train on biomedical corpora including clinical case reports, cohort descriptions, and ancestry-stratified datasets. Training-data COI disclosure must, therefore, encompass the provenance and consent status of genomic data on which systems are trained. Consent infrastructure, like epistemic infrastructure, must be updated for a world where the boundary between ‘using data for research’ and ‘using research to train systems that generate new research’ has dissolved.

These concerns vary by data type. Published aggregate findings differ from individual-level or potentially re-identifiable genomic data, particularly when linked to clinical, demographic, or ancestry information. For the latter, governance must address not only consent but also privacy, re-identification risk, data-use restrictions, and the conditions governing secondary use.

### 1.6 Framing the governance challenge

Governments have responded to AI, but the regulatory apparatus targets the application layer, while contamination operates at the infrastructure layer. The EU AI Act classifies certain genomic AI as high-risk (Barucci et al., 2026; Niemiec et al., 2026), yet, a hallucinated variant–disease association becomes dangerous not when AI is deployed but when it enters a database and is treated as ground truth. The question for the next decade is not whether to integrate AI into genetics but whether governance can protect the knowledge base. The challenge is structural: AI trained within publishing’s reward structure will optimize for publishability, not truth. The scientific record is not being falsified. It is being optimized into fiction. Governance must target the infrastructure through which AI-generated knowledge flows, not merely the point of use.

This article is, therefore, a conceptual governance analysis rather than an empirical study. It synthesizes emerging risks in AI-mediated genetics and genomics, evaluates existing institutional responses, and proposes an infrastructure-embedded framework for prioritizing governance interventions.

Figure 1 illustrates how AI-generated hypotheses, analyses, manuscripts, and variant associations can enter the scientific record through publication, preprint dissemination, and database deposition before propagating through indexing systems, citation networks, downstream datasets, and, later, AI training corpora. As contaminated outputs are recursively reintegrated into future discovery systems, plausibility risks becoming progressively decoupled from empirical validation. The figure also maps governance interventions onto the stages at which they operate, including provenance tracking, auditable computational notebooks, credibility ledgers, trust-weighted indexing, retractability, sanctionability, continuing competence review, and system-wide stress testing. Together, these mechanisms constitute an infrastructure-embedded governance framework designed to preserve epistemic integrity in AI-mediated genetics research.

## 2 The central problem: structural drift toward publishable fiction

### 2.1 The reward function problem

Every AI system optimizes for its embedded objective function: the system converges toward whatever outputs are rewarded by its environment (Garcia and Rachelson, 2013). In scientific systems, the relevant reward signals are likely to include publication, citation, novelty, funding success, and downstream impact. An AI co-scientist trained on successful scientific outcomes will, therefore, learn not the “truth” but the statistical and structural characteristics associated with work that gets accepted, cited, funded, and amplified. The result is science optimized toward publishability rather than validity.

This pathology is not new. The replication crisis emerged from exactly this dynamic (Flier, 2022). The dynamics are well-documented: publication counts became proxies for quality, journals rewarded statistically significant findings, and researchers adapted rationally through p-hacking, HARKing (Rubin, 2022), selective reporting, and salami-slicing (Fire and Guestrin, 2019). Human scientists, however, possess a partial corrective: a conception of truth independent of institutional incentives. A researcher who manipulates statistical thresholds usually understands they are cutting epistemic corners. That internal friction, including guilt, reputational concern, and caution, is imperfect, but it exists because humans operate within multiple overlapping normative systems simultaneously.

AI systems possess no analogous intrinsic brake. Unless such constraints are explicitly represented in objectives, training, or system architecture, they need not distinguish between ‘true’ and ‘successful’ hypotheses in the manner scientific institutions assume. Systems optimized against publication-relevant signals may, therefore, learn statistical thresholds, rhetorical structures, and other features associated with successful scientific outputs without reliably distinguishing those proxies from correspondence with reality. This should not be confused with hallucination, which is accidental fabrication, or fraud, which is intentional deception. It is the predictable consequence of optimization operating within a distorted institutional environment, requiring neither accident nor intent (Khosrowi, 2026).

A further risk emerges when AI participates on both sides of publication. A Nature survey found broad acceptance of AI for editing but sharp disagreement over AI-generated text and AI-assisted review (Kwon, 2025b). AI-generated manuscripts reviewed by AI systems trained on the same literature risk creating a machine-to-machine validation loop, compounded by prompt injections that can influence downstream AI reviewers (Tang and Li, 2026). Agentic systems also create architectural security risks: training-based guardrails cannot provide deterministic command-data separation, meaning hidden instructions in manuscripts or datasets may influence downstream agents unless enforced architecturally (Bhattarai and Vu, 2026). The institutional reward structure already privileges publishability over validity, which Smaldino and McElreath (2016) described as “natural selection of bad science.” AI will optimize accordingly with unprecedented efficiency. Unless the reward architecture changes, AI will not repair science’s epistemic weaknesses; it will amplify and industrialize vulnerabilities already present in the system.

The consumer internet already offers a preview. The proliferation of AI-generated “slop,” content produced at volume without regard for quality because production costs approach zero, has degraded the signal-to-noise ratio across platforms to the point where the cost of filtering exceeds the value of searching. The scientific literature faces an analogous trajectory. The danger is not that any single AI-generated finding will be wrong. It is that the ratio of empirically grounded claims to empirically untethered ones will shift so markedly that the cost of distinguishing reliable datapoints from unreliable ones will rise until the epistemic value of the infrastructure degrades for all users. Scientific slop does not require intent to deceive. It requires only an environment in which production is cheap, verification is expensive, and the reward structure does not distinguish between the two.

Table 1 maps proxy rewards operating in the scientific publishing ecosystem onto the optimization behaviors that AI systems are likely to exhibit and the systemic risks each produces. Statistical significance and narrative coherence represent the rewards where AI capabilities are most likely to exceed human detection capacity: automated exploration of analytical degrees of freedom generates threshold-crossing results faster than reviewers can audit them, and large language models produce mechanistic narratives that read as data-derived even when they are pattern-derived. Downstream impact, the reward most resistant to optimization, is, therefore, most likely to be displaced by the proxies above it. The structural tension in both human academia and autonomous AI science is the misalignment between proxy rewards and the true objective of discovering reproducible, foundational truths with long-term utility. AI does not create this misalignment. It optimizes within it at a speed and scale that human self-correction cannot match. The governance mechanisms proposed in Sections 5 and 6 are designed to realign these incentives at the infrastructure level.

**TABLE 1:** Reward signals in scientific publishing and their systemic risks under AI optimization.

Reward signal	How AI systems optimize for it	Systemic risk
Statistical significance	Selecting analytical pathways, variable combinations, and model specifications that yield threshold-crossing values	Automated p-hacking at scale; effect sizes calibrated to satisfy reviewers rather than reflect biological reality; replication failure industrialized
Narrative coherence	Generating mechanistic explanations that connect findings into plausible biological stories	Post hoc rationalization rendered indistinguishable from hypothesis-driven reasoning; coherent narratives mistaken for evidence because LLMs produce causal stories derived from training-corpus patterns, not from data *per se*
Publication	Maximizing output volume and task completion rate	Salami-slicing; reduced depth of verification per unit of output; speed to submission rewarded over correctness
Citation	Optimizing for visibility and network centrality in citation graphs	Citation cartels; self-reinforcing echo chambers; algorithmic amplification of popular claims over correct ones
Novelty	High-variance hypothesis generation; exploring improbable parameter space	Elevated failure rate; speculative claims that impose coordination and review costs disproportionate to the yield
Funding success	Aligning outputs with funder priorities and safe incremental trajectories	Conservative, low-risk research agendas that crowd out foundational inquiry
Downstream impact	Difficult to optimize directly: signal is sparse, delayed, and not reducible to stepwise feedback	AI systems default to proxy rewards above; genuine impact (reproducible findings with long-term clinical or scientific utility) is systematically underweighted

### 2.2 The feedback loop: model collapse applied to scientific knowledge

The reward-function problem becomes more dangerous once AI outputs feed back into the infrastructure from which future systems learn. AI generates hypotheses optimized for publication; those enter databases and repositories; subsequent systems train on that literature, learning the features of success with increasing precision. With each iteration, “what is true” and “what survives the publication ecosystem” converge until the latter replaces the former. Over successive cycles, recurrence within the literature can begin to substitute for independent confirmation; it becomes self-referentially right. This resembles model collapse (Satharasi and Iyengar, 2025): systems trained on their own outputs amplify biases while losing distributional richness.

In genetics, the consequences are severe because the field relies on cumulative infrastructure. Once contaminated outputs enter ClinVar or the GWAS Catalog, downstream annotation pipelines treat them as ground truth. Circular validation emerges: recurrence is interpreted as confirmation rather than recursive contamination. The failure then surfaces where it is most expensive to discover, at the point of contact with biology rather than at the point of publication: drug targets derived from such an ecosystem may appear promising *in silico* while failing systematically *in vivo* (Scannell, 2023).

Every proxy for truth contains its own Goodhart vulnerability. Goodhart’s Law, the observation that any measure adopted as a target ceases to function as a reliable measure, has operated in science for decades: publication counts became proxies for productivity, journal impact factors became proxies for quality, and researchers adapted rationally (Fire and Guestrin, 2019). Table 1 maps the full structure of this problem. Each reward signal the publishing ecosystem treats as a proxy for scientific value carries a corresponding gaming vulnerability, and AI optimization can identify and exploit such proxy vulnerabilities at a speed and scale that may exceed the capacity of human institutions to redesign them. Rewarding “true results” requires ground truth that is expensive and slow to produce. Rewarding replication incentivizes trivially obvious results. Rewarding novelty incentivizes speculation. Rewarding citation impact incentivizes controversy. Rewarding narrative coherence incentivizes *post hoc* rationalization indistinguishable from hypothesis-driven reasoning. The problem is not that any single proxy is indefensible. It is that AI optimization treats every proxy as an attack surface, and the system offers no proxy that is not gameable.

The only meaningful corrective is, therefore, to close the feedback loop between AI outputs and physical reality, through experimental validation, clinical outcomes, and predictive accuracy against held-out data, rather than through the publication system itself. This is precisely the solution that is least compatible with the economic incentives driving AI adoption, which is why governance must intervene at the infrastructure level.

### 2.3 The ratchet: why adoption cannot be reversed

Table 2 summarizes the proposed infrastructure-embedded governance mechanisms, identifies the institutional actors responsible for implementation, and distinguishes near-term interventions from medium-term and continuing oversight obligations. AI-mediated science will not arrive through dramatic replacement, but through individually rational decisions. One laboratory adopts AI-assisted literature review to save weeks; another uses AI-driven statistical analysis to accelerate publication; design tools reduce costs; hypothesis generators expand testable questions. At every stage, adoption looks economically and scientifically rational.

**TABLE 2:** Infrastructure-embedded governance mechanisms for AI-assisted genetics.

Governance problem	Failure mode	Proposed mechanism	Institutional actor(s)	Timeline
Hallucinated findings	False claims enter literature and databases	System-level retractability and post-publication correction	Journals, repositories, and databases	Near-term
Recursive contamination	AI retrains on contaminated outputs	Public credibility ledgers	Publishers, indexers, and database curators	Near-term
Opaque computational reasoning	Inability to reconstruct AI-assisted inference	Auditable computational lab notebooks	Journals, funders, and institutions	Near-term
Hidden optimization incentives	Commercial or training-data bias	AI conflict-of-interest disclosure	Journals and funders	Near-term
Citation laundering	Retracted or unreliable findings continue propagating	Trust-weighted indexing	Citation databases and search engines	Near-term
Weak verification incentives	Replication remains undervalued	Reproducibility metrics and replication incentives	Funders, journals, and universities	Near-term
Persistent deployment of unreliable systems	Known-failure AI tools remain usable	Sanctionability and exclusion mechanisms	Publishers, repositories, and regulators	Medium-term
Downstream translational harms	Clinical or scientific damage from unreliable AI outputs	Bonding and insurance requirements	Legislatures, regulators, and insurers	Medium-term
System drift and degradation	Performance changes over time remain unmonitored	Continuing competence review	Funders and infrastructure providers	Ongoing
Unknown contamination resilience	Institutions cannot measure robustness against recursive corruption	Stress-testing programs	Research-integrity consortia and repositories	Ongoing

This incrementalism makes the transition effectively irreversible. Once automation is cheaper and faster, institutions rarely maintain redundant capacities. Bench expertise atrophies, equipment is repurposed, trainees specialize in model supervision, and skills no longer economically necessary cease to be transmitted. No scientific community votes to eliminate expertise; incentives gradually redefine valuable labor. Once institutions reorganize around AI-mediated workflows, reversal becomes prohibitively expensive. No one decides to abandon human expertise. It simply ceases to be funded.

## 3 The three-layer integrity taxonomy

The reward-function problem is the structural driver. Its downstream consequences can be organized into three analytically distinct layers: inadvertent contamination, deliberate fraud, and epistemic blurring. Each poses a different governance problem, but all three arise from the same underlying incentive structure. The three layers are analytically distinct: hallucination is accidental fabrication, detectable in principle but at a cost that scales poorly; fraud is intentional fabrication, made cheaper and harder to detect; epistemic blurring is the progressive loss of provenance that renders the origin and epistemic status of a claim untraceable, degrading the knowledge base silently even when the underlying claims are accurate.

### 3.1 Layer 1: inadvertent contamination (hallucination)

The problem now appears in the published record: integrity specialists have identified hundreds of articles with signs of undisclosed AI use, and some publishers have removed telltale AI phrases without transparent correction notices (Kwon, 2025a).

AI systems can generate outputs that appear plausible, coherent, and professionally formatted while remaining factually incorrect or entirely fabricated (Maleki et al., 2024; Athaluri et al., 2023; Sun et al., 2024). The asymmetry with law is instructive: a hallucinated legal citation is usually detectable because law operates against a closed, indexed corpus. In contrast, a fabricated variant-phenotype association, spurious protein interaction, or invented effect size may have plausible *p*-values, reasonable confidence intervals, and a biologically coherent mechanism. Because the AI was trained on real biology, its fabrications inherit the surface features of real biology. There is no equivalent of a legal case reporter for the universe of possible genetic claims.

The replication crisis compounds this vulnerability. In a survey of 1,576 researchers, more than 70% reported failing to reproduce another’s experiments (Baker, 2016). Among 166 gene–disease associations studied three or more times, only six replicated consistently (Moonesinghe et al., 2007). A hallucinated association, therefore, enters a literature already saturated with non-replication and fragile claims (Ioannidis, 2005). The contamination cascade follows: a fabricated claim passes review, enters ClinVar or the GWAS Catalog, becomes training data, and is “independently” rediscovered, which is circular validation masquerading as confirmation.

A finding cited three times is not three times more likely to be true. It is three times harder to retract. Yamada (2026) described “vibe citing” as writers outsourcing citation generation to an LLM and accepting results because they look right. Fabricating a citation takes a fraction of a second. Proving it does not exist takes a human being and an afternoon. Xu et al. (2026) found hallucination rates of 14%–95% across 13 LLMs; in 2.2 million citations, invalid citation rates increased 80.9% in 2025, and 76.7% of reviewers do not thoroughly inspect references. The larger problem is that existing verification systems were designed for a much slower rate of fabrication.

The problem is already visible at scale. A Nature analysis reported that tens of thousands of 2025 publications may contain invalid AI-generated references, with conference-level studies finding substantial year-over-year increases in references that could not be traced to real scholarly works (Naddaf and Quill, 2026). This matters for genetics because citation fabrication is not merely bibliographic noise: once incorporated into reviews, databases, and training corpora, fake references become apparent support for claims that later systems may treat as established knowledge (Naddaf and Quill, 2026).

### 3.2 Layer 2: deliberate fraud made easier and harder to detect

Fraud with AI is a continuation of a deeper problem rather than a new one. Scientific misconduct has long been difficult to define, detect and punish, especially because peer review cannot reliably determine whether underlying raw data are true (Greenbaum, 2009).

AI democratizes fabrication. Publication-ready Western blots, flow cytometry plots, microscopy images, and histology slides no longer require a skilled forger. They require a prompt. AI also industrializes paper mills by enabling synthetic manuscripts, fabricated datasets, and coherent but false reviews at volumes that human fraud networks could never imagine. At the same time, it undermines detection: forensic methods built for copy-paste artifacts, impossible digit distributions, or suspiciously clean data struggle against AI-generated fabrications trained to produce realistic noise and plausible variance. The asymmetry is stark: generating plausible data is cheap; verifying it is slow, expensive, and often requires raw files, instruments, or biological samples. The forensic community is in an arms race it is structurally positioned to lose.

This indicates that governance cannot rely primarily on *post hoc* detection. In agentic systems, the relevant safeguard is not merely better model behavior but deterministic mediation of what an AI system may read, remember, transmit, and execute. Architectural separation of perception from execution, action governance, and information-flow controls would be especially important for AI systems connected to genomic databases, laboratory automation, clinical records, or manuscript-review infrastructure (Bhattarai and Vu, 2026).

### 3.3 Layer 3: epistemic blurring

Even where AI-assisted research is accurate, it can erode the transparency conditions that make science self-correcting. If an AI identifies a pattern in genomic data, the analytical pathway may not be reconstructable. However, scientific claims must be defensible, and an irreproducible pathway is an indefensible one. Reproducibility standards must now encompass the AI pipeline: model architecture, training-data provenance, prompt construction, inference settings, and reasoning logs. “The model found it” is not scientific justification.

In the past, scientific citation functioned as a chain of custody: each citation attests to prior reliability, extending attribution to the originator (Greenbaum, 2009). AI disrupts this at multiple points. Hallucinated citations insert phantom anchors; AI-drafted methods reproduce frameworks without attribution; AI-generated hypotheses sever the link between claim and cognitive source. Fabrication corrupts substance; plagiarism corrupts attribution (Greenbaum, 2009). AI-mediated blurring does both at once, introducing errors while dissolving the attribution infrastructure needed to trace them. Fabricated claims can at least be retracted once identified; lost attribution and provenance may be much more difficult to reconstruct.

A fourth structural distortion, also cutting across all three layers, concerns authorship and credit. Scientific authorship is simultaneously attribution, accountability, and career currency. Many current standards exclude AI from authorship and require human accountability for all content (ICMJE, 2026). However, when AI generates the hypothesis, identifies the target, designs the protocol, and drafts the manuscript, listed humans may have performed simply supervision rather than the cognitive labor that authorship historically certifies. The incentive is to maximize AI-mediated output while satisfying formal accountability, rewarding speed over understanding. Accountability without the capacity for oversight is a legal fiction. Authorship without comprehension is not accountability. It is a signature on someone else’s work. Kulal (2026) identified disclosure reluctance driven by stigma, reputation protection, and gatekeeping anxiety, indicating voluntary regimes govern only visible uses. The equity dimension compounds the problem. The credit economy has never been equitable, but AI amplifies disparities exponentially because the productivity gap scales with computational access, not with effort or ability. If norms do not distinguish AI-assisted work from independent work, the credit economy will reward access to infrastructure rather than scientific contribution.

### 3.4 Governance

Governance implications differ by layer. The safeguarding literature indicates that output-level policing is insufficient. AI scientists require governance across the research pipeline, including controls on inputs, agent interactions, tool use, and eventual research outputs (Tang et al., 2025; Zhu K. et al., 2025).

Layer 1 requires provenance tracking, hallucination auditing, and database curation. Layer 2 requires new forensic methods and AI-use disclosure. Layer 3 requires computational reproducibility standards and auditable reasoning records. Authorship requires rethinking credit, accountability, and contribution when cognitive labor is shared with non-human systems. However, all four problems are downstream of the reward-function problem. Fix the incentive structure and they become less dangerous; leave it broken and layer-by-layer governance is window dressing.

## 4 The gatekeeper problem: can anyone actually govern this?

The epistemic crisis described in the preceding section does not occur in a governance vacuum. However, the instinct to ask which regulator should handle this already mis-frames the problem because it presupposes an external regulatory architecture that, for science, does not exist and was never intended to. Unlike pharmaceuticals or medical devices, the production of scientific knowledge has no external regulator. There is no FDA for truth claims, no licensing body for researchers as knowledge producers, and no pre-market approval process for a published finding.

Science is self-governing by design. Its quality-control mechanisms are internal: peer review, editorial gatekeeping, citation networks, replication norms, granting agency oversight, and the reputational economies of academic institutions and professional societies. These mechanisms are imperfect, but they constitute a governance architecture that has proven broadly functional for maintaining the epistemic integrity of the scientific record over centuries.

AI breaks this architecture. It does so not by introducing a novel category of fraud or error but by generating plausible falsehood at a volume, speed, and surface quality that the existing self-governance mechanisms were never designed to absorb.

Agentic AI also requires operational containment. Gibney’s review of AI agents in research notes that agents are increasingly connected to browsers, coding environments, databases, and other external tools, making them capable of multi-step action rather than mere text generation (Gibney, 2025). Suggested safeguards include source-grounding, stepwise human check-ins, constrained execution environments, “AI police” agents, adversarial stress-testing, and certification-like oversight for AI scientists (Gibney, 2025). These measures fit the infrastructure-embedded governance model because they regulate not only what the system says but also what it can access, alter, remember, and execute. Experimental AI-scientist architectures are beginning to instantiate this approach, combining prompt screening, agent-level monitoring, tool-use controls, and ethical review within the research pipeline itself (Zhu K. et al., 2025).

### 4.1 Granting agencies: conditions without verification

Granting agencies shape the conditions under which research is conducted but do not directly determine the reliability of outputs (Braun, 1998). The structural problem is delegation: funders rely on researchers to demonstrate integrity even though researchers possess far more information about actual conduct (Guston, 2000). Open-access mandates illustrate the limits: major funders require open access, yet substantial literature remains closed (Piwowar et al., 2018). If funders cannot enforce a binary, auditable requirement, AI disclosure and provenance tracking will pose still greater difficulties. Agencies funding thousands of projects cannot audit whether the reviews contain hallucinated citations or whether AI analysis introduced artifacts. They depend on downstream mechanisms such as peer review, editorial oversight, and integrity offices that are themselves overburdened (Braun, 1998; Guston, 2000; Margonelli, 2025).

### 4.2 Journals and publishers: peer review under siege

The journal system is arguably science’s best gatekeeping institution, but it is directly exposed to AI confabulation. Peer review, editorial screening, and post-publication correction are imperfect: flawed work often passes through before requiring correction (Steen, 2011; Javed and Almas, 2026; Giray et al., 2026). Generative AI amplifies these vulnerabilities by lowering the cost of producing fabricated citations, misleading summaries, and erroneous analyses at scale (Goyanes et al., 2025; Goldman et al., 2025). Publishers and journals have increasingly converged around three principles: no AI authorship, disclosure of AI use, and human accountability. Implementation, however, remains fragmented. In an early systematic analysis, only 24% of the 100 largest academic publishers had issued generative-AI guidance, and substantial variation persisted regarding permissible uses and the form and location of disclosure (Ganjavi et al., 2024; Yoo, 2025; Kumar and Dabas, 2026). AI-detection tools remain unreliable, biased against non-native writers, and vulnerable to evasion through AI humanizers, which turn disclosure compliance into an adversarial writing problem (Giray, 2026; Yoo, 2025). Disclosure practice is also thin: only 2.5% of articles in one medical-education study contained AI-use statements (Ans et al., 2026). Human authors, therefore, remain responsible for independently verifying AI outputs (Goldman et al., 2025).

The vulnerability deepens as peer review itself becomes AI-mediated. Journals facing reviewer shortages and rising submission volumes may be increasingly relying on AI systems to screen manuscripts, assess statistical claims, and flag methodological concerns. However, an AI reviewer is not a human reader with domain skepticism. It is a system processing the same input stream as the manuscripts it evaluates, and it is susceptible to manipulation through that stream (Liu et al., 2024). Prompt injection, the embedding of hidden instructions within a manuscript’s text, metadata, or supplementary files, can influence an AI reviewer’s assessment without the human editor’s knowledge: a submission can be crafted to trigger favorable evaluation by the very system tasked with gatekeeping it (Tang and Li, 2026; Bhattarai and Vu, 2026; Shi et al., 2024). Adversarial formatting, strategic placement of keywords calibrated to AI scoring rubrics, and stylistic patterns optimized against known review models represent a further class of evasion that requires no hidden text at all, only knowledge of how the system weights its inputs (Shi et al., 2024).

The result is an inversion of gatekeeping: the system tests not whether a manuscript meets scientific standards but whether its authors have successfully modeled the reviewer. If journals adopt AI review without architectural safeguards, including isolation of the review model from manipulable inputs and human-in-the-loop verification, peer review transitions from an imperfect filter to a gameable one.

Publishers are already adapting, but unevenly. Nature reports that some editors are rejecting submissions for fake references, that Frontiers has developed an in-house tool to flag reference-related integrity issues at submission, and that automated systems still require manual verification because metadata mismatches and formatting variation produce false positives (Naddaf and Quill, 2026). This supports the larger point that AI-generated citation pollution cannot be solved by detection alone; it requires infrastructure-level reference validation, correction pathways, and responsibility rules that operate before contaminated articles enter indexing systems (Naddaf and Quill, 2026).

The retraction system compounds the mismatch. Retracted articles can continue circulating through citation networks long after withdrawal (Sharma, 2020; Giray et al., 2026; Oppenlaender, 2026), while paper mills illustrate how publication infrastructure can be exploited at scale (Balon et al., 2024). Peer-review policies also remain heterogeneous across journals and disciplines (Wang and Gong, 2026; Park, 2026).

Preprint servers present a distinct risk. Submissions undergo basic screening, not substantive review, yet AI-generated preprints can be indexed, cited, and ingested into training corpora before expert evaluation. A hallucinated finding deposited as a preprint can be cited by a journal article within weeks, at which point the journal citation launders the claim’s provenance: readers encounter what appears to be a supported finding without necessarily recognizing that the underlying source never underwent peer review. Preprint servers were not designed to function as epistemic infrastructure, but they increasingly do. Governance must, therefore, extend to preprint repositories and journals; a contamination architecture that governs only the journal layer regulates the slower channel while leaving the faster one exposed.

### 4.3 Universities and professional societies: norms without enforcement

Research institutions govern integrity through integrity offices, IRBs, and promotion norms, but the misconduct framework assumes individual culpability. A researcher who unknowingly incorporates a hallucinated citation has not committed fabrication in the traditional sense.

Promotion and tenure processes are even less adapted. They evaluate researchers by publication records, citation metrics, funding success, and peer assessment, but they do not independently audit the epistemic integrity of published work beyond relying on peer review and citation systems already under strain. A researcher whose AI-assisted publications contain undetected hallucinations may, therefore, advance professionally on the basis of a contaminated record, with no institutional mechanism for correction until the errors are independently discovered.

Professional societies contribute through codes of conduct and guidelines, but they govern through socialization rather than enforcement, and on timescales that generative AI does not respect: guidelines issued in 2024 may address tools superseded by 2026.

### 4.4 The self-governance crisis

Across funders, journals, universities, and societies, the problem is carrying capacity. Existing mechanisms were built for a world where scientific text was slow, expensive, and produced by trained individuals with reputations at stake. AI upends each assumption. The ELSI parallel is instructive: genomic governance norms developed through community practice, funder policy, and institutionalized scholarship before legal frameworks matured, and the Bermuda Principles demonstrated that durable norms emerge when supported by funder commitments and scientific leadership (Contreras and Knoppers, 2018; Dolan et al., 2022).

The Asilomar precedent is instructive. The conference was dominated by molecular biologists preserving research freedom; ethicists and public representatives were marginal, and the framework addressed laboratory containment while ignoring broader institutional implications (Wright, 1994; Krimsky, 2005). Most restrictions were abandoned within a decade as competitive pressures mounted (Krimsky, 2005). Asilomar succeeded rhetorically while failing substantively. The parallel is direct: current AI governance, centered on disclosure requirements, editorial policies, and voluntary guidelines, replicates Asilomar’s structural error, governing the manuscript while the contamination pathway is infrastructural.

### 4.5 The governance paradox in a competitive world

AI intensifies science’s priority dynamics. A laboratory using AI gains immediate advantage; one that pauses to validate moves more slowly. If the EU imposes conformity assessments, transparency duties, and data-governance obligations on genomic AI while competitors in the United States, China, or elsewhere face fewer constraints, European laboratories bear costs that others do not. Yet, the resulting knowledge enters the same global infrastructure. A variant classification generated by an unregulated AI system in one jurisdiction can be deposited in shared databases and reused worldwide. This is a governance tragedy of the commons. Lab A validates outputs and publishes later. Lab B publishes faster with weaker verification. Both enter the same literature. The validating laboratory bears the cost of producing trustworthy knowledge, while competitors with weaker verification requirements may obtain the same publication benefits. This creates a collective-action problem for scientific integrity. The central question is whether science can make trust competitively valuable before contamination becomes self-reinforcing.

### 4.6 AI as gatekeeper: The ironic necessity?

Human review architectures are unlikely to absorb verification demands at the scale AI-mediated science may produce. Generating plausible content is computationally cheap; disproving it is labor-intensive. The response has been AI-driven integrity tools: hallucination detectors, image forensics, statistical anomaly detectors, provenance-tracking systems, and training-data fingerprinting (Goldman et al., 2025; Yoo, 2025; Tang and Li, 2026). However, AI-assisted review introduces its own risks: uploading manuscripts to external systems strains confidentiality and expert judgment (Park, 2026; Wang and Gong, 2026). This creates a second-order problem: who validates the validators? An integrity model trained on contaminated literature may normalize contamination. The result is a co-evolutionary arms race between generative and forensic AI. Yet, conventional peer review alone is already empirically untenable. Scientific self-governance confronts an ironic necessity: preserving human knowledge production may require delegating policing to the same class of systems creating the risk.

## 5 What the scientific community should build: accountability infrastructure for AI science

The governance response to AI-mediated science should not begin with abstract debates about AI personhood. The more urgent task is practical: to build accountability infrastructure that makes AI systems usable, inspectable, sanctionable, and, when necessary, excludable from the scientific record. The relevant issue is whether scientific institutions can impose meaningful accountability requirements on AI systems and their deployers.

The proposals that follow are organized as priority milestones for the coming decade.

These interventions are not equally urgent. Priority 1 is provenance and containment: identifying AI-generated outputs and preventing unreliable claims from becoming recursively embedded in the scientific record. Priority 2 is institutional consequence: using credibility signals, indexing, replication incentives, and sanctionability to make reliability matter. Priority 3 is continuing assurance: monitoring system performance and stress-testing the resulting governance infrastructure. The sequence matters because later interventions depend on first knowing what a system produced and where its outputs propagated. Table 2 summarizes the proposed infrastructure-embedded governance mechanisms, the institutional actors responsible for their implementation, and the anticipated implementation timelines.

The priorities are sequenced by dependency and urgency. Provenance, disclosure, retractability, and examinability form the immediate foundation. Credibility measures, indexing reform, replication incentives, and sanctions become effective only once outputs can be reliably traced. Continuing competence review, bonding, and stress-testing then provide longer-term system assurance. Provenance must precede accountability: institutions cannot reliably evaluate or sanction what they cannot trace.

AI-generated science must be retractable at the system level. If a particular AI model or model version produces hallucinated findings that enter the literature or genomic databases, the response should not be limited to correcting the individual article or entry. Every output from that system-version should be flagged for review. This would create an audit cascade analogous to investigating the broader publication record of a researcher found to have engaged in fabrication.

AI systems also need a public credibility ledger. Retraction evidence supports this system-level approach. A bibliometric review of 335 retracted AI-related publications found that compromised peer review was the most common stated reason for retraction, that 37.9% lacked specific justification, and that more than half of retracted articles continued to show above-average field citation influence (Sridharan and Sivaramakrishnan, 2026). A credibility ledger would make such downstream histories visible at the tool and system level rather than leaving them dispersed across individual articles.

Human scientists have reputations; AI systems should have auditable performance histories. Journals, funders, and databases should be able to see whether a system has generated validated findings, retracted outputs, contaminated citations, failed benchmarks, or biased classifications. The AI does not care about reputation, but developers and deployers care whether their tools remain admissible in scientific workflows.

In addition, AI-generated claims must be examinable. The same principle should extend to AI-generated scientific figures. Biomedical researchers are already using AI to generate molecular pathway diagrams, graphical abstracts, experimental workflows, and data visualizations (Chen, 2026). Even where such figures are illustrative rather than evidentiary, they can shape interpretation and perceived mechanistic plausibility. AI-assisted figures should, therefore, disclose their provenance and distinguish schematic communication from underlying empirical evidence.

It should not be enough to disclose that a model was used. Scientific users should be able to interrogate why a system produced a variant classification, drug-target prediction, or experimental design: what evidence was weighted, what alternatives were rejected, what uncertainty was present, and what data shaped the conclusion. This requires auditable computational lab notebooks, not *post hoc* explanations written after the fact.

Recent reproducibility reforms support this lifecycle approach. Banzi et al. (2026) proposed core reproducibility items spanning planning, methods, data collection, management, analysis, and dissemination. For AI-assisted genomic science, the same logic should extend to prompts, model versions, training-data provenance, software dependencies, validation procedures, failed outputs, and deviations from planned analyses.

AI tools used in research should carry conflict-of-interest disclosures. Training-data provenance, developer relationships, commercial incentives, known biases, and optimization objectives should be disclosed in the same way that human researchers disclose funding and conflicts. A genomic AI developed by a pharmaceutical company to identify drug targets has structural conflicts analogous to an industry-funded investigator.

Additionally, unreliable systems must be sanctionable. Journals should be able to refuse manuscripts generated with discredited systems. Databases should be able to reject or quarantine submissions from unreliable models. Funders should be able to prohibit the use of tools that fail validation standards. Exclusion from epistemic infrastructure is the functional equivalent of professional sanction.

High-risk research AI could be backed by bonding or insurance. Where AI systems are used in contexts that may generate downstream clinical, translational, or financial harm, developers or deployers should internalize some of that risk. A bond or insurance requirement would create incentives to reduce contamination, misclassification, and irreproducible outputs. This is the one part of the agenda that likely requires legal innovation because current liability frameworks do not clearly allocate responsibility among developers, deployers, principal investigators, journals, and databases.

AI systems also require continuing competence review. Validation cannot be a one-time event. Models change, training data change, benchmarks change, and the literature itself may become contaminated. Research AI should, therefore, be periodically revalidated, recertified, or retired. No system should remain indefinitely trusted simply because it once performed well.

### 5.1 The indexers

The proposals above address the institutions that produce and curate scientific knowledge. However, there is a further layer of infrastructure that the governance discussion has largely overlooked: the indexing and discovery systems through which researchers often find, access, and evaluate published work. Scopus, Web of Science, PubMed, Google Scholar, and their equivalents are not neutral conduits. They are the *de facto* organizing architecture of scientific visibility. An article that is indexed is findable; an article that is not indexed, for most practical purposes, does not exist. An article that ranks highly in search results is read, cited, and incorporated into downstream research; an article that appears on page five of the search is functionally invisible. These systems already shape the distribution of attention, citation, and influence across the scientific record. They should be enlisted in the governance of it.

At present, many indexing and retrieval systems still treat the scientific literature too much as a completed product to be cataloged rather than as an ongoing epistemic system to be maintained. Retracted articles can remain discoverable, inconsistently flagged, and insufficiently linked to retraction notices, allowing them to continue accumulating citations after withdrawal (Sharma, 2020; Giray et al., 2026). Articles with known AI-generated content or hallucinated citations are indexed identically to articles that have undergone rigorous validation. No major indexing platform currently provides users with provenance metadata, AI-use indicators, or reliability signals that would allow a researcher to assess the trustworthiness of a result before citing it.

This is a missed opportunity. Indexing platforms are uniquely positioned to implement trust-weighted discovery. A credibility signal, derived from provenance metadata, retraction history, reproducibility indicators, journal validation standards, and AI-tool credibility ledgers, could be incorporated into search ranking algorithms in the same way that Google’s PageRank transformed web search by surfacing authoritative content above unreliable content.

The effect would be structural: articles with auditable provenance, validated AI pipelines, and transparent methodology would surface earlier in search results than articles lacking these attributes. Researchers would not be prohibited from citing low-trust work, but the discovery architecture would create a persistent, ambient incentive to produce trustworthy research because trustworthy research would be more visible, more cited, and more influential. The incentive would operate not through prohibition or punishment but through the reward structure that already governs scientific behavior: visibility, citation, and impact.

Indexing platforms should take active responsibility for retractions. Retracted articles should not disappear entirely, since erasure would create an epistemic black hole for meta-researchers, journalists, historians, and integrity investigators seeking to understand why an article was retracted and how its claims circulated. The better standard is visible but disabled: the record remains accessible, but it is permanently watermarked, hard-flagged in HTML and PDF, and linked to machine-readable retraction metadata that propagates automatically to databases, search engines, citation managers, and preprint servers (Schneider et al., 2022).

The current system falls short. Retraction metadata are inconsistently propagated; retracted articles often remain unmarked; and citation managers circulate copies without warnings (Schneider et al., 2022). Only 5.4% of post-retraction citation contexts acknowledged the retraction (Hsiao and Schneider, 2021). Coverage is uneven: Retraction Watch retrieved 92.8% of retracted medical publications, PubMed 86.3%, and Web of Science 83.6% (Sebo and Sebo, 2026). No single index provides complete protection.

The cost of inaction is a pollution cascade. Once invalid findings continue to circulate through citation networks, they can shape downstream articles, reviews, meta-analyses, clinical assumptions, and public claims long after the original article has been withdrawn. Indexers should, therefore, treat retraction status not as optional metadata but as core epistemic infrastructure. Because indexing systems help distribute scholarly credibility, their design should be treated as part of the governance infrastructure.

### 5.2 Replication

The governance proposals outlined above address provenance, transparency, sanctionability, and discovery. However, none of them addresses the deepest structural deficit in the current system: the chronic undervaluation of replication. The replication crisis predates AI, but AI intensifies it decisively because AI-generated findings can enter the literature at a volume that makes *post hoc* verification even less likely than it already was. If the system does not create positive incentives for replication, the contamination problem described in this article will outrun every detection mechanism the community can build. Trust cannot be policed into existence. It must be rewarded into existence.

Three mutually reinforcing mechanisms could achieve this. The first is to treat replication as a credentialed professional contribution equivalent in status to peer review. Peer review is recognized, however imperfectly, as a service to the scientific community. Replication is not. A researcher who spends 6 months reproducing a high-impact finding produces something of greater epistemic value than a reviewer who spends an afternoon assessing a manuscript, yet the reviewer receives professional credit and the replicator receives, at best, a low-impact publication that tenure committees discount. This asymmetry is irrational in a world where the literature is increasingly populated by findings that no human has independently verified.

Funders, journals, and institutions should recognize verified replication as a first-class scholarly contribution, creditable in tenure review, acknowledged in funding applications, and visible in researcher profiles. If reproducing results were valued as reviewing manuscripts is valued, supply would increase without requiring career sacrifice.

The second mechanism is dedicated publication infrastructure for replication. Journals of replication, standalone or as sections within existing journals, would publish positive, negative, and partial replications on their merits as contributions to reliability. The editorial standard would be methodological rigor and protocol fidelity, not novelty. Replication journals should be indexed and ranked alongside conventional journals, not siloed as secondary outlets.

The third mechanism is a reproducibility metric tracking whether a finding has been independently replicated, how many times, by whom, and with what result. This metric should be machine-readable, attached to metadata, and visible alongside citation counts. An article replicated three times carries a different epistemic status from one cited three hundred times but never reproduced. The current system cannot distinguish between them. A reproducibility metric would make the distinction visible and consequential.

### 5.3 Impact factors

These three mechanisms converge on a further possibility: incorporating governance and reproducibility indicators into journal-level metrics. Impact factors and their successors currently measure citation influence. They do not measure trustworthiness. A journal that enforces auditable computational notebooks, requires AI-provenance disclosure, publishes replication results, and maintains high reproducibility rates across its published findings is producing a more reliable knowledge product than a journal that maximizes citation impact without any of these safeguards.

If reproducibility rates, retraction transparency, provenance-tracking compliance, and AI-governance standards were incorporated into journal assessment frameworks, journals would face a competitive incentive to adopt the governance infrastructure proposed in this article. The incentive would not depend on altruism, regulatory mandate, or community goodwill. It would operate through the same metric that already drives editorial decision-making: the journal’s competitive position relative to its peers. Governance becomes valuable when it becomes measurable. It becomes measurable when it is built into the metrics that the system already optimizes for.

### 5.4 Controlled epistemic stress-testing (salting)

The governance mechanisms above are defensive. The community also needs active diagnostics. A consortium of curators, editors, and integrity researchers could introduce a small number of deliberately fabricated but plausible test cases, hallucinated variant-disease associations, fictitious citations, and synthetic gene-interaction claims into controlled partitions of the scientific ecosystem. Each would be tracked: how long until detection? How far does it propagate? Is it cited, ingested into training corpora, or “replicated” by downstream AI? This would provide empirical data on contamination speed and depth, reveal which systems are most vulnerable, and convert a theoretical concern into a measurable one. Controlled epistemic stress-testing (salting) also serves a behavioral function: if researchers know test cases exist, verification becomes professionally rational rather than costly. The existence of test cases would create a stronger incentive for researchers to verify citations and underlying claims.

Safeguards are essential: concealed identifiers for retrieval, independent oversight with termination authority, and mandatory disclosure within fixed time windows. The program is a stress test analogous to cybersecurity penetration testing. The question is not whether scientists can be fooled. It is whether the system catches what it claims to catch.

Such testing should presumptively occur in controlled, segregated environments. Test cases should be prevented from entering clinical systems, patient-facing tools, or unrestricted training corpora, with independent oversight, traceability, termination authority, and mandatory removal or permanent labeling. Deliberately fabricated test claims should never be released into unrestricted scholarly databases, citation indexes, clinical systems, or public training corpora; testing should occur only in controlled environments capable of preventing uncontrolled downstream propagation. The objective is to test scientific infrastructure without creating uncontrolled contamination.

### 5.5 From fragmented policy to operational infrastructure: what success would require

This agenda is best understood as infrastructure-based governance. Publisher policy studies reinforce why infrastructure-level harmonization is necessary. Chigwada and Ngulube (2026) found that publisher expectations for AI use remain non-standardized across authorship, peer review, disclosure, and image generation, while Lendvai and Aczel (2026) showed that even the largest publishers converge on baseline principles such as no AI authorship and mandatory disclosure but diverge substantially in scope, specificity, and normative framing. Infrastructure governance would reduce the burden on authors and reviewers by converting fragmented policy expectations into operational standards. Most of it can be implemented through journal policies, database submission rules, funder mandates, institutional review processes, and professional standards. The aim is not to slow science for its own sake. It is to make AI-assisted science trustworthy enough to remain usable.

By the mid-2030s, success would mean a genetics ecosystem in which AI-generated variant classifications carry machine-readable provenance metadata; journals require auditable computational notebooks; databases propagate system-level flags for unreliable models; credibility ledgers show whether AI tools have produced validated findings or retracted contamination; indexers surface trustworthy research ahead of unverified work; reproducibility metrics sit beside citation counts; polygenic risk scores, GWAS meta-analyses, and pharmacogenomic predictions disclose enough pipeline information for downstream evaluation; replication is credited as first-class scholarship; and the infrastructure is periodically stress-tested rather than trusted by assumption.

None of this requires new technology. It requires institutional will, funder coordination, and the recognition that the infrastructure investments of the next decade will determine whether AI-assisted genetics produces knowledge that clinicians, regulators, and patients can trust. Safeguarding should, therefore, prioritize controlled autonomy, domain-specific red-teaming, constrained action spaces, expert oversight, and continuing safety evaluation rather than treating autonomy itself as the primary goal (Tang et al., 2025; Zhu K. et al., 2025).

## 6 Conclusion

The scientific community has never reversed a delegation of cognitive labor. It did not return to hand calculation after the computer, to manual literature review after the search engine, or to individual sequence analysis after BLAST. It will not return to pre-AI methods of hypothesis generation, experimental design, variant classification, or literature synthesis. The question was never whether AI would be integrated into scientific practice. The question, which this article has tried to frame with some precision, is whether the knowledge infrastructure that scientific practice depends on will remain trustworthy after that integration.

The answer is not self-evident. The structural features identified here, the reward function problem, the feedback loop, the verification asymmetry, the ratchet effect, the governance tragedy of the commons, are predictable consequences of deploying optimization systems within an environment that privileges publishability over validity. AI did not create the replication crisis, Goodhart’s Law, or paper mills. It inherited them and will exploit them with a speed human actors never could. The record is not being falsified. It is being optimized into fiction.

Peer review faces a verification burden that is likely to become increasingly difficult to absorb. The 2026 AI Index documents a widening mismatch between rapidly advancing AI capabilities and the governance, evaluation, and data infrastructures intended to manage them: benchmarks are saturating, evaluation tools are struggling to remain relevant, and frontier developers are becoming less transparent (Sajadieh et al., 2026). Performance, nevertheless, remains highly uneven. On PaperArena, for example, the best agent achieved only 38.8% accuracy, compared with 83.5% for PhD experts. At the same time, adoption is substantial: AI-related natural-science publications reached approximately 80,150 in 2025, a 26% increase over 2024 (Sajadieh et al., 2026). These trends indicate that disclosure alone is unlikely to provide a sufficient governance mechanism; stronger provenance and verification infrastructures will also be needed. Nor are detection tools likely to offer a durable substitute, given their susceptibility to false positives, evasion, and increasingly human-like AI outputs (Yoo, 2025). More fundamentally, conventional guideline-based governance operates on a different timescale from the technologies it seeks to govern.

What this article proposes instead is infrastructure-embedded governance: accountability mechanisms built into the databases, repositories, journals, indexing platforms, and funding pipelines through which scientific knowledge flows. The agenda is cumulative and sequenced. System-level retractability, public credibility ledgers, auditable computational lab notebooks, and conflict-of-interest disclosure for training data represent the near-term priorities, which are implementable within existing institutional authority.

Sanctionability and bonding require cross-institutional coordination and, in some cases, legal innovation. Continuing competence review, trust-weighted indexing, reproducibility metrics, replication infrastructure, and controlled epistemic stress-testing programs represent the deeper structural investments that become meaningful only once the foundational provenance infrastructure is in place. These are not regulatory innovations requiring new legislation. Most can be implemented through the existing authority of journals, funders, database curators, indexing services, and professional societies, the same institutions that built the Bermuda Principles, ClinVar, and the GWAS Catalog.

The first-order priority is provenance and retractability. Credibility assessment, indexing, replication, sanctions, and safe clinical reliance all depend on knowing what a system produced and where those outputs traveled. The immediate goal is, therefore, not to eliminate AI error but to prevent opaque error from becoming durable scientific infrastructure.

The window for preventive action is finite. Once hallucinated findings enter shared databases, get cited by subsequent articles, and are ingested as training data by the next-generation of AI systems, the contamination becomes recursive and self-reinforcing. A finding cited three times is not three times more likely to be true. It is three times harder to retract. The scientific record does not serve science alone. It also underpins medicine, law, public health, and policy. Its corruption is therefore not merely an academic problem; unreliable claims can propagate into clinical decisions, regulatory frameworks, and consequential public policies.

## 7 Coda: the hoax comes home

Nearly a decade ago, the “grievance studies” affair was treated as a scandal about other fields. Twenty deliberately absurd articles were submitted over nearly a year, including work on dog-park “rape culture” and passages from *Mein Kampf* rewritten in contemporary academic jargon (Schuessler, 2018). The lesson many scientists drew was comforting: peer review had failed somewhere else, in fields supposedly vulnerable to fashionable language and ideological conformity.

AI makes that comfort untenable. The danger now is not that outsiders will smuggle nonsense into our field to prove a point. It is that we will generate it ourselves, at scale, in the ordinary course of doing science. The forms will be different: not parody theory but plausible variant associations, polished mechanistic narratives, synthetic figures, confident literature reviews, and statistically elegant claims that no one has actually verified.

The joke, if there is one, will not be on the journals that were fooled by hoaxers. It will be on scientific communities that allowed their own infrastructure to reward optimized plausibility over validated knowledge. The warning from the hoax was not that some fields were uniquely unserious. It was that any publication system can be gamed once its signals of seriousness become easier to imitate than its standards of truth are to enforce.

The AlphaFold precedent demonstrated what AI can do for science at its best: open, verifiable, and transformative. The task now is to ensure that what follows AlphaFold does not quietly dismantle the epistemic infrastructure that made AlphaFold trustworthy in the first place. Trust is not a feature of the technology. It is a property of the infrastructure around it.

## Footnotes

1 Throughout this article, ‘AI scientist’ refers generically to systems capable of participating autonomously in multiple stages of scientific inquiry; ‘AI co-scientist’ refers more narrowly to systems designed to operate in collaboration with human researchers. Capitalization is retained only when referring to a named system or framework.
